# Development and Training of Mindfulness and Its Relationship with Executive Function in Chinese Rural Upper-Grade Elementary School Students

**DOI:** 10.3390/bs15040422

**Published:** 2025-03-26

**Authors:** Sihui Zheng, Bin Zhang, Haichun Zhou, Mingyi Wang

**Affiliations:** 1 School of Humanities and Social Sciences, Beijing Forestry University, Beijing 100083, China; zhengsihui@bjfu.edu.cn (S.Z.); m18333167189@163.com (B.Z.); 2 Graduate School of Education, Peking University, Beijing 100871, China; zhouhaichun2023@stu.pku.edu.cn

**Keywords:** mindfulness, executive function, rural elementary school student

## Abstract

The purpose of this study was to explore the development and relationship between mindfulness and executive function (EF) in students in grades 4–5 and investigate mindfulness training’s impact on mindfulness and EF in grade 4 students in rural China. Study 1 measured mindfulness and EF in Chinese students in grades 4–5. Differences in the level of mindfulness among children in grade 4 (*n* = 159) and grade 5 (*n* = 187) in rural China were analyzed by multi-factor analysis of variance. Correlation and hierarchical multiple regression analyses were used to explore the relationship between mindfulness and EF in grade 4 (*n* = 103) and grade 5 (*n* = 124). Study 2 included 45 4th graders with a general mindfulness level, with 21 children in the experimental group receiving 12 mindfulness training lessons. The mindfulness and EF scores of individuals in the mindfulness and control groups were tested before and after the intervention. Study 1 showed that 4th graders had significantly lower mindfulness scores than 5th graders. EFs in grades 4–5 were significantly correlated with mindfulness. Study 2 revealed that mindfulness training significantly improved the experimental group’s mindfulness and working memory. A non-significant improvement in inhibitory control and cognitive flexibility was observed. There was a significant difference in mindfulness in grades 4 and 5 of Chinese rural upper-grade elementary school. Children who perform well in mindfulness also perform well in EFs. Mindfulness training improved the mindfulness and working memory of 4th graders in rural China with a general mindfulness level.

## 1. Introduction

Mindfulness is conscious awareness and acceptance of the present moment. It involves two core processes: first, to sustain attention to the current experience, and second, to accept and refrain from judging anything that occurs in the moment (Kabat-Zinn, 2003). Mindfulness, the awareness of paying attention to the inner state of the present moment, changes with children’s growth, and its development is related to enhanced self-awareness (Brown & Ryan, 2003). Mindfulness has positive effects on individual development and is a protective factor against adversity (Zapolski et al., 2019; Zhou et al., 2017). Dispositional mindfulness was noted to be positively associated with optimistic views of the past and the future and was closely linked to happiness (Erguler et al., 2023; Khoury, 2023). Research has shown that mindfulness is an internal developmental asset for adolescents, with one longitudinal study finding that fourth graders who experienced lower risk reported higher mindfulness in the seventh grade and that both risk and asset factor levels were associated with mindfulness in the seventh grade. This finding suggests that trait mindfulness may develop as a function of adolescents’ ecological normative experiences in daily life (Warren et al., 2021). Understanding the development of mindfulness and its relationship with various other functions could help scholars comprehend the development and role of mindfulness more thoroughly.

Mindfulness training, which emphasizes focusing on one’s attention and learning to be open to acceptance, has been widely used in various fields of intervention and is equally important for minors (López-Ramón et al., 2023; Lutz et al., 2008). Studies have shown that mindfulness-based interventions significantly reduce rumination among students in grades 5–8 (Butterfield & Roberts, 2022). A meta-analysis suggested that school-based mindfulness interventions could increase prosocial behavior, resilience, attention, and mindfulness in students and reduce anxiety and attention problems (Phan et al., 2022). Moreover, mindfulness interventions have a reliable effect on students’ academic performance (Verhaeghen, 2023). Mindfulness training has also demonstrated protective effects, with research noting that for children facing adversity, mindfulness facilitates cognitive and emotional regulation and supports social and school success (K. Diamond, 2024).

Executive function (EF) is a psychological process that enables individuals to consciously control their thoughts and actions and is a vital indicator of cognitive function (H. Li & Wang, 2004). EF influences children’s academic performance, emotional regulation, and social interactions and plays a significant role in their cognitive development and personality improvement (Riggs et al., 2015; Shan & Zhou, 2021; C. Wang, 2019). Miyake et al. (2000) have treat working memory, inhibitory control, and cognitive flexibility as the three core subcomponents of EF, and all of these three components develop during childhood (Zelazo & Carlson, 2020). Working memory refers to the ability to hold information temporarily in the mind and process it in the mind depending on the situation (A. Diamond, 2012). Around the age of 10, there is a period of rapid development of working memory (Gathercole et al., 2004). Inhibitory control refers to an individual’s ability to inhibit the distraction of irrelevant information and actively control maintaining one’s attention on a specific object (Zelazo, 2002). The ability of inhibitory control develops relatively early. It tends to stabilize in the middle and upper grades of elementary school, yet it is not fully mature. It can still be enhanced due to individual differences and training (Davidson et al., 2006). Cognitive flexibility is an individual’s ability to adapt to different conditions and switch between different environments (A. Diamond, 2013). Cognitive flexibility gradually increases to the adult level at the age of 15 (Huizinga et al., 2006). The three subcomponents of EF are interrelated yet independent, potentially yielding distinct training effects.

Research has found that rural children perform significantly worse than urban children on various EF tests (Tine, 2014; Wijeakumar et al., 2019). This may be associated with the lower socioeconomic status (SES) of families among rural children. SES, a major environmental factor influencing individual development, has a profound impact on individual development. Negative factors that are detrimental to children’s cognitive development, such as excessive stress, cognitive deprivation, and low parental involvement in parenting, tend to exist in families with low levels of SES (Evans & Kim, 2013; Ziegler et al., 2020). Studies have found that the gap in cognitive performance associated with differences in high and low levels of family socioeconomic status persists into an individual’s adulthood (Last et al., 2018). In recent years, China’s economy has been developing rapidly, but rural children’s family socioeconomic status is still generally lower than that of urban children (Chen et al., 2020). This may predict poorer future development for rural children, such as lower income as well as poorer health and higher crime rates in adulthood (Blair & Razza, 2007; Oshri et al., 2019). Therefore, exploring practice-heavy EF approaches has important implications for the future development of rural children in China.

Mindfulness is closely linked to EF. An analysis of the brain mechanism of trait mindfulness revealed that individual differences in mindfulness were significantly related to the precuneus, which is associated with high-level cognitive functions related to self-awareness (Yeager et al., 2022). Holas and Jankowski (2013) argued that mindfulness is a type of meta-awareness that involves and cultivates EFs and attention. Thus, EF can be used simultaneously with practicing mindfulness. Several studies have shown that mindfulness training affects EFs (e.g., Crowe & McKay, 2016). According to the Liverpool model of mindfulness, mindfulness is influenced by cognitive flexibility and emotion regulation (Malinowski, 2013), and individuals with high inhibitory control are also better able to adopt positive mindfulness as a mode of action (Lee & Chao, 2012). Mindfulness and EFs play complementary roles. The link between mindfulness and healthier psychological states may be due to mindfulness reflecting higher-order cognitive processes related to EFs and other behavioral and emotional processes (Lyvers et al., 2014). Linking mindfulness to EFs, examining their relationship, and finding effective ways to enhance children’s EFs are important for children’s cognitive development. However, few studies have examined the development of mindfulness in children and its relation to cognitive development. Research has indicated that students in grades 7–8 with well-developed mindfulness also have better working memory and inhibition control (Riggs et al., 2015). Geronimi et al. (2020) reported that working memory, inhibition, and cognitive flexibility were significantly associated with mindfulness in elementary school children aged 7–13 years. However, the cited study included a large age span, and age should be divided into more detailed and specific groups for analysis. Additionally, the study inadequately specifies the relationship between mindfulness and distinct EF components, particularly within rural child populations.

In recent years, mindfulness training has been widely applied in training research in the field of cognition, such as EFs, and has demonstrated significant positive effects (Ng et al., 2023). Most research has shown that the three core components of EFs (working memory, cognitive flexibility, and inhibitory control) in adults and adolescents can be improved by mindfulness training (Ahmed Aboalola, 2024). Mindfulness can promote the development of EFs and activate cognitive functions related to emotion regulation and behavioral control (Lyvers et al., 2014). Monitor and Acceptance Theory (MAT) posits that mindfulness training enhances cognitive functioning, particularly executive functioning, through two core mechanisms: monitoring and acceptance (Lindsay & Creswell, 2017). The monitoring process relies on an executive attention network. By repeatedly anchoring attention to the present moment (e.g., breath, sensory experiences), mindfulness practice strengthens selective and sustained attention, thereby reinforcing brain regions associated with executive functioning (A. Diamond, 2013; Tang et al., 2015). Concurrently, the nonjudgmental acceptance of distracting stimuli (e.g., intrusive thoughts, emotions) reduces cognitive resource depletion from emotional regulation, freeing resources for information retention and processing (Jha et al., 2019). For working memory, mindfulness training increases activation in the dorsolateral prefrontal cortex (DLPFC), a core neural substrate of working memory by repeatedly anchoring attention (e.g., breath-focused practices) (Tang et al., 2015; Jha et al., 2010). For inhibitory control, mindfulness may improve conflict sensitivity through attention monitoring by enhancing anterior cingulate cortex (ACC) activity (Tang et al., 2015), which is critical for conflict detection, whereas its modulation of basal ganglia inhibitory circuits requires prolonged training (Lutz et al., 2008). Cognitive flexibility, reliant on the dynamic shifting of attentional focus (e.g., task rule changes) and coordinated activation of the prefrontal–parietal network (Dajani & Uddin, 2015), can be effectively trained by integrating mindfulness with targeted task-switching exercises (e.g., Wisconsin Card Sorting) (A. Diamond & Lee, 2011). However, a meta-analytic study concluded that the benefits of mindfulness training on EFs are specific and complex rather than general (Gallant, 2016). Some studies argue that brief mindfulness sessions (e.g., 15-min interventions) yield no significant benefits for EFs, including inhibitory control, cognitive flexibility, or working memory (Hartanto et al., 2023; Quek et al., 2021). This likely reflects that brief mindfulness interventions fail to induce the immediate neurofunctional adaptations necessary to enhance higher-order cognitive processes. A study involving 9–10-year-old children showed that mindfulness training did not improve “cold” inhibition in a neutral environment to control the neutral environment (Rezende et al., 2023). A meta-analysis highlights that mindfulness exhibits the smallest effect size on cognitive flexibility compared to its impacts on working memory and inhibitory control (Cásedas et al., 2020). These findings collectively suggest that mindfulness training may exert gradual influences on executive functioning, particularly for higher-order processes like inhibitory control and cognitive flexibility. According to MAT, monitoring is improved prior to reception in mindfulness training (Lindsay & Creswell, 2017). The core of working memory is the online retention and updating of information, which requires a continuous focus on task-relevant stimuli (Cowan, 2001), and mindfulness training basically trains working memory through the monitoring pathway. Inhibitory control, on the other hand, requires the inhibition of dominant responses and involves conflict acceptance processes (Miyake et al., 2000). Cognitive flexibility requires the dynamic switching of task rules, and the acceptance process can help individuals to quickly disengage from the current task and reduce cognitive rigidity (A. Diamond, 2013). Therefore, mindfulness training may prioritize the strengthening of working memory.

For minors, studies have also found positive effects of mindfulness on EFs. A study of 6th and 8th graders revealed positive effects of a mindfulness intervention on improvements in EFs (Lassander et al., 2020). Thierry et al. (2016) used a randomized experimental design to conduct a school-based early childhood mindfulness program with 47 children (*M* = 4.57, *SD* = 0.27) in the southwestern United States. The results revealed that at the end of the first year of the project, the program participants demonstrated improvements in teacher-reported EF skills, particularly those related to working memory, planning, and organization. Flook et al. (2010) used a randomized controlled trial of mindfulness training with 72 children aged 7–9 years to validate the effectiveness of mindfulness training on EFs. At the same time, this study found that the improvement in EFs by mindfulness training worked in children with poor EFs, suggesting that mindfulness training may have a meaningful effect on the improvement in EFs in rural elementary school students. But this study did not specify the effects of different components. A U.S. randomized controlled trial evaluated a 6-week school-based intervention for preschool children from low-income families, comprising three experimental arms: Mindfulness+Reflection training, Literacy training, and Business-as-Usual instruction. The results revealed that the mindfulness-based pedagogical intervention did not yield superior EF skill improvements compared to Literacy training. However, it demonstrated promising EF enhancements relative to Business-as-Usual during follow-up assessments weeks after the intervention, suggesting its potential utility for supporting EFs’ development in low-income preschoolers (Zelazo et al., 2018).

Although studies have found that mindfulness training is effective in improving the attention of rural elementary school students (Ricarte et al., 2015), no study has yet systematically explored the role of mindfulness training on the EFs of rural elementary school students. Culturally, mindfulness relies on specific contextual knowledge, intentions, and aspirations and cannot be actualized in a culture-free manner (Kirmayer, 2015). Eastern traditions (e.g., Buddhist meditation) emphasize nonjudgmental introspection (Kirmayer, 2015), whereas Western mindfulness interventions (e.g., MBSR) prioritize structured cognitive training (Lutz et al., 2008). This divergence may contribute to cultural specificity in mindfulness’s effects on EFs. Educationally, parents at lower social levels are more likely to fall into an automated negative response mode rather than positive parenting due to traditional parenting concepts or time pressures, which may lead to low levels of positive parenting in children (Whittingham, 2016). Children in rural areas exhibit delayed EF development compared to urban peers due to insufficient educational resources (e.g., lack of psychology curricula, inadequate teacher training) and limited cognitive stimulation at home (e.g., scarcity of books, intellectually enriching activities) (Freitas et al., 2022; Noble et al., 2015); mindfulness interventions in such contexts are required to address larger cognitive deficits. This study found that more positive feedback on mindfulness training came from Asian children, female participants, and those with lower economic status (Shi, 2024). Research on mindfulness training among rural Chinese children not only elucidates intervention mechanisms in low-SES and culturally distinct settings but also offers critical empirical insights for cross-cultural comparisons (e.g., collectivist vs. individualist cultural orientations) and educational stratification studies (e.g., urban–rural disparities).

In summary, there are important reasons to discuss how mindfulness training in rural children can contribute to fostering outcomes. The period between the ages of 10 and 13 represents a critical stage in the development of EFs among elementary school students. For rural children with relatively poor EF levels, this developmental window offers a particularly opportune time for the enhancement of EF capabilities. Among such ages of students, those in grade 4 demonstrate significantly lower levels of EF than those in grade 5 do, which represents a critical phase for EF development (Y. Wang et al., 2012). Thus, it is worthwhile to study whether mindfulness training can increase the level of EFs and its subcomponents among children at this crucial stage of development.

To address the above two problems, two studies were designed to gain a clearer understanding of the relationship between mindfulness and EFs in elementary school students in grades 4–5 and to verify the effectiveness of mindfulness training in improving EFs. Study 1 analyzed the development and relationship between mindfulness and EFs among 4th- and 5th-grade rural elementary school students by measuring their levels of mindfulness and EFs, and to provide a theoretical foundation for intervention research in Study 2. Study 2 included rural elementary school students in grade 4 with a general level of mindfulness to examine whether mindfulness training improved their mindfulness and EFs. Based on the existing research, we established two hypotheses. First, the mindfulness level of 4th- and 5th-grade students in rural Chinese elementary schools demonstrates developmental progression and exhibits significant correlations with both overall EF and its constituent components. Second, mindfulness training significantly improved working memory for children in grade 4 of rural elementary schools, and the working memory of children in the experimental group significantly improved after the mindfulness intervention relative to the control group. However, given that inhibitory control and cognitive flexibility involve more complex training pathways and neural functions, mindfulness training may not significantly enhance the level of inhibitory control and cognitive flexibility in rural Chinese elementary school students with lower overall resource levels. The present study innovatively examines the development of mindfulness in rural elementary school students and the relationship between mindfulness and EFs. It also explores the impact of mindfulness training on EFs and its various subcomponents in rural elementary school students during the critical period of EF development. Based on previous research (Flook et al., 2010; Q. Li et al., 2019), this study referred to the core concepts of mindfulness training, improved previous programs based on the cognitive development characteristics of elementary school students, and designed a set of mindfulness training programs suitable for students’ development. This study’s results could provide direct causal evidence of the effect of mindfulness training on improving the mindfulness and EFs of elementary school students in rural China and have positive practical implications for enriching mindfulness training programs for various age groups.

## 2. Study 1

### 2.1. Materials and Methods

#### 2.1.1. Participants

To explore the developmental trend of mindfulness, a MANOVA with G-power was used to determine the sample size, where *α* = 0.05 and *p* = 0.95 were selected. The final calculated sample size was 280. A total of 346 elementary school students (174 boys and 172 girls) aged 9–12 years (*M* = 10.66, *SD* = 0.89) of grades 4–5 of 3 elementary schools in rural China were randomly selected to measure the level of mindfulness. Among them, 159 students were in grade 4 (*M* = 10.16, *SD* = 0.75) and 187 were in grade 5 (*M* = 11.10, *SD* = 0.76). All students were from rural families. Their parents’ occupations are part-time or farming, and their education level does not exceed tertiary education. To explore the correlation between mindfulness and EFs, we used correlation analysis. G-power was used to determine the sample size, based on Cohen’s (2013) definition of statistical efficacy and with reference to previous studies (Bai et al., 2021), medium effect sizes were selected (*r* = 0.30, *α* = 0.05), and the effect was tested (*p* = 0.80). The calculated sample size was 82. Since many children’s parents go out to work, only 227 students (107 boys and 120 girls) had their parents fill out the EF questionnaire. Subjects were missing at random, and there was no significant difference in the level of mindfulness between the set of 227 subjects and that of 346 subjects (*t* < 0.01, *p* = 1.00). Of the total of 227 students aged 9–12 years (*M* = 10.44, *SD* = 0.90), 103 were in grade 4 (*M* = 9.93, *SD* = 0.77) and 124 were in grade 5 (*M* = 10.86, *SD* = 0.77). The research adhered to the principles outlined in the Declaration of Helsinki, and ethical approval was granted by the Institutional Committee of Science and Research Ethics.

#### 2.1.2. Procedure

The data were collected when the students were in school. Students and their parents were informed about the main objectives of the study, inclusion criteria, ethical standards, and data protection policies when accessing the link through an informed consent form. All of them were told that their participation was voluntary, anonymous, and confidential. Only participants who agreed to the study conditions were able to participate in the research. The children were subsequently invited to complete the Mindful Attention Awareness Scale, the parents were invited to complete the Behavior Rating Inventory of Executive Function, and the data were entered and recorded.

#### 2.1.3. Measures

- Measurement of children’s mindfulness. The Mindful Attention Awareness Scale for Children (MAAS-C) was constructed by Lawlor et al. (2014) based on the Mindful Attention Awareness Scale (MAAS; Brown & Ryan, 2003). The revised scale contains 15 questions in total. A 6-point Likert scale with positive scores is used, and subjects’ higher test scores correspond to stronger mindfulness. The average age considered in the past was 10.16 years, and the internal consistency coefficient was 0.84 (Schonert-Reichl et al., 2015). This measurement of mindfulness in children has good reliability and validity. Subsequently, Y. Wang et al. (2021) revised MAAS-C. The revised scale demonstrated Cronbach’s alpha coefficient of 0.87, indicating good reliability, and confirmed its applicability for rural children. The Cronbach’s *α* coefficient of this scale in this study was 0.81 and 0.82.
- Measurement of children’s EF. The Behavior Rating Inventory of Executive Function (BRIEF) was developed by Gioia et al. (2000) and is suitable for children aged 6–18 years. The scale has 86 questions and is divided into two dimensions: behavior management and metacognition. The former includes three subdimensions: inhibition, shifting, and emotional control. The metacognitive dimension includes initiation, working memory, planning, organization, and monitoring. A three-point scale (1 = never; 2 = sometimes; 3 = often) with reverse scoring was used. The higher the test score was, the lower the level of EF. This study used the Chinese version revised by Qian and Wang (2007). The internal consistency coefficient of the revised scale ranged from 0.74 to 0.96, which made it suitable for the measurement of EF in Chinese children. The Cronbach’s *α* coefficient of this scale in this study was 0.96.

#### 2.1.4. Data Analyses

To examine the differences in the levels of mindfulness and EFs between the 4th and 5th grades, a multi-factor analysis of variance was conducted. Grade, gender, and age were included as independent variables, and the score of mindfulness or EFs was included as dependent variables in order to analyze differences in them across grade after controlling for gender and age. To examine the relationship of mindfulness and EFs, grade was set as the fixed variable, the MAAS-C scores were set as the dependent variable, and age and gender were set as the control variables. A correlation analysis and a hierarchical multiple regression were performed on the scores of mindfulness and EF of all participants. To control for the gender, age, and school of the children, in the hierarchical multiple regression, mindfulness scores were used as the independent variable, gender and age were used as the first stratum, and EF and all of its dimension scores were used as the second stratum dependent variables. The above analyses were conducted to examine the relationship between mindfulness and EFs among rural elementary school students in grades 4–5. All data analyses were performed using the Statistical Package for Social Sciences (SPSS, version 27.0).

### 2.2. Results

The homogeneity of variance test was not significant (*p* > 0.05), and it could be analyzed by an analysis of variance. The descriptive statistics and results of the analysis of variance for the levels of mindfulness and EF are presented in Table 1. The results of the multi-factor analysis of variance for mindfulness indicated that grade was significant (*F*(1, 344) = 13.03, *p* < 0.01). There was a differential relationship in mindfulness levels between the 4th and 5th grades, with the mindfulness level of 5th-grade children being significantly higher than that of 4th-grade children. Neither gender (*F*(1, 344) = 2.39, *p* = 0.12) nor age (*F*(3, 342) = 2.15, *p* = 0.14) was significant, suggesting that there were no significant relationships in these aspects with the total score.

The results of the multi-factor analysis of variance for EF showed that the grade difference was not significant (*F*(1, 225) = 0.45, *p* = 0.50), indicating that there was no differential relationship in the level of EF between the 4th and 5th grades. The control variables, gender (*F*(1, 225) = 1.13, *p* = 0.29) and age (*F*(3, 223) = 0.06, *p* = 0.81), were also not significant, suggesting that there were no differential relationships between these factors and the EF scores.

The descriptive statistics of EFs, each dimension score, and an analysis of its correlation with the mindfulness score are displayed in Table 2. The results revealed that mindfulness scores were significantly and negatively correlated with the total EF and its subdimension scores. Since higher BRIEF scores indicate lower levels of EFs, the results suggest that higher levels of mindfulness are associated with higher levels of EFs.

The results of hierarchical linear regression are presented in Table 3. After controlling for gender, age, and grade level, significant linear associations were observed between mindfulness and both the overall EF score and its subcomponents, including emotional control, working memory, initiation, material organization, shifting, planning, monitoring, and inhibition among 4th- and 5th-grade students. Consequently, after accounting for the effects of gender and age, mindfulness demonstrated significant linear relationships with EF and all its subdimensions.

## 3. Study 2

### 3.1. Materials and Methods

#### 3.1.1. Participants

Based on the school and teacher’s class schedule, the number of students participating in the training was limited. Therefore, this study synthesized the opinions of teachers and schools to screen the children who participated in the training. According to the children’s mindfulness scores in grade 4 (*M* = 63.74, *SD* = 11.82), those who scored 75 or more had relatively strong mindfulness (with mindfulness scores above the sum of the mean and one standard deviation), and mindfulness training may not be meaningful for them. In contrast, children with average mindfulness performance may be better able to benefit from short-term training (Creswell et al., 2014). Study 2 used a 2 × 2 control group and experimental group, pretest and post-test design with a sample size budget using G-power, choosing a medium effect size of *f* = 0.25, *α* = 0.05, and a test effect of *p* = 0.8. The calculated sample size was 44. Forty-eight children with mindfulness scores below 75 in grade 4 of an elementary school in Jing County, Hengshui City, China, aged 10–11 years (*M* = 10.33, *SD* = 0.59) were selected for training and randomly divided into experimental and control groups. Children in both experimental and control groups were explicitly instructed to refrain from discussing the contents of the intervention, thereby preventing contamination of the research outcomes. Three students in the former were unable to complete the mindfulness training due to leaves of absence and other reasons, and hence the final number of students who participated in the experiment was 45. The age range of the subjects was 10–11 years (*M* = 10.44, *SD* = 0.50), and the size of the experimental group was 21 (10 males and 11 females), with an age range of 10–11 years (*M* = 10.29, *SD* = 0.45). The control group included 24 participants (12 males and 12 females) aged 10–11 years (*M* = 10.58, *SD* = 0.49). The number of subjects recruited for this study was 45, which was able to fulfill the requirements of the experiment.

#### 3.1.2. Procedure

This study adopted a 2 × 2 experimental and control group with a pretest and post-test design. Neither the experimental group nor the control group had been exposed to mindfulness-related exercises before training. The students in the experimental group were given mindfulness training courses for 40 min each four times a week for three weeks. They were taught by graduate students in psychology, who were psychology professionals trained to teach the courses according to the requirements. Children in the experimental group were convened in dedicated classroom settings at weekly intervals for mindfulness training. Students in the control group participated in regular classes. Mindfulness and EFs, including working memory, inhibitory control, and cognitive flexibility, were measured before and after training by classic tasks. In particular, inhibitory control was subdivided into two parts for measurement: response inhibition and interference inhibition. The measurement tools for the pretest and post-test were the same. The pretest and post-test were administered at the beginning and at the end of the week of the course, respectively, by the authors of the paper together with the school teacher. After the experimental group had completed the mindfulness training, the researcher gave the relevant training materials to the school teacher, who then administered the mindfulness training to the children in the control group. The research adhered to the principles outlined in the Declaration of Helsinki, and ethical approval was granted by the Institutional Committee of Science and Research Ethics.

#### 3.1.3. Measures

The Mindful Attention Awareness Scale for Children (MAAS-C) was used, and the scale was the same as in Study 1. The Cronbach’s α of this scale in this study was 0.72.

As experimental research needs to focus more on internal validity, this study selected an experimental paradigm of EFs suitable for 4th-grade children to measure the subdimensions of children’s EFs based on previous research. The selected experimental paradigms were all commonly used measurement tasks for each subdimension of EF.

- Number recitation task. The present study measured children’s working memory via a number recitation task. A computer screen showed successive numbers, and children needed to present the numbers in the reverse order of their appearance after all the numbers had been presented. During this time, the children were not allowed to make sounds. For example, if a child saw the numbers 2-3-5, he or she would need to report 5-3-2 and press ‘Enter’ to move to the next trial. The difficulty of the task started from two numbers, and there were three trials for each difficulty level. Children needed to complete at least two trials to move to the next difficulty level. The ‘+’ interface at the center of a computer screen was presented for 500 ms, and a stimulus was presented for 1000 ms. The experiment was divided into 6 practice trials (breadth 2–3) and 21 experimental trials (breadth 3–9). Scoring was based on the number of correct answers given by the child (Prencipe et al., 2011).
- Stop signal task. This study used an adapted version of the classic stop signal task to measure children’s response inhibition ability. The experiment was divided into Go trials and Stop trials. The probability of Go trials was 75%, and the frequency of Stop trials was 25%. In the former, when a white arrow pointing in any direction appeared on the computer screen, the subject responded by pressing a key according to the direction of the arrow. In Stop trials, no response was to be made when the stop signal (a small blue triangle above the arrow) appeared, regardless of which side the arrow was on. The formal experiment was divided into two blocks, each containing 70 trials, and the stop signal reaction time and the Stop trial’s accuracy were recorded (Y. Wang et al., 2020). In the present study, two elementary metrics were employed to assess task performance: (1) the stop signal reaction time (SSRT) was calculated by determining the temporal interval between each response stimulus and the stop signal, specifically the mean Stop Signal Delay (SSD). The SSRT for each participant was derived by subtracting the mean SSD from the Go trial’s reaction time. A longer SSRT indicates poorer response inhibition capability. (2) The accuracy rate of Stop trials represents the proportion of successful inhibitions when a stop signal is presented.
- Stroop task. This study used the Stroop task to measure children’s interference inhibition. The task stimuli were words in red, yellow, green, and blue. The subjects judged the colors of the words presented and pressed the corresponding buttons. The measurement task was divided into two trials: color–word congruence and color–word incongruence. Being congruent means that the color of a word is consistent with its meaning (e.g., the word ‘red’ being displayed in red). Being incongruent means that the color of a word is not consistent with the word’s meaning (e.g., the word ‘red’ being shown in green). The congruent and incongruent trials accounted for 50% of the total number of trials. The formal experiment was divided into two blocks containing 70 trials (Y. Wang et al., 2020). This study used the difference between the reaction times of congruent and incongruent trials to express the performance on the task. The smaller the difference was, the better the subject’s interference inhibition.
- Letter case switching task. This study used a letter case switching task to measure children’s cognitive flexibility. Letters of different colors appeared on the computer screen. The children judged the upper and lower cases according to the color of the letters and pressed the corresponding keys. The formal experiment was divided into two blocks containing 70 trials. The reaction time and accuracy rate were recorded as indicators (Ren, 2018).

#### 3.1.4. Data Analyses

The EFs and mindfulness scores of the two groups of students were measured, and the resulting data were collected. First, independent sample t tests were performed on the total EFs score, EF subcomponent scores, and mindfulness scores of the pretests of the experimental and control groups to ensure the homogeneity of the groups. Subsequently, an analysis of covariance (ANCOVA) was conducted to examine the mindfulness levels between the two groups of students, with pretest mindfulness scores, gender, and age included as covariates for control purposes. Third, the study investigated the effects of mindfulness training on each subdimension of EFs. ANCOVA was employed to examine the effects of the mindfulness intervention on inhibitory control, cognitive flexibility, and working memory. For inhibitory control and cognitive flexibility, reaction time served as the elementary outcome measure. The group assignment was treated as the independent variable, while the post-test reaction times were designated as the dependent variable. Pretest reaction times, post-test accuracy rates, gender, and age were included as covariates to control for potential confounding factors. Parallelism tests were performed beforehand. The collected data were analyzed using SPSS 27.0.

#### 3.1.5. Intervention Outline

This study used the concept of mindfulness training, combined with the research of Flook et al. (2010) and Q. Li et al. (2019), based on the cognitive development level of elementary school students and the three stages of mindfulness awareness (Weare, 2013) to develop a mindfulness training program using mindfulness games. During the relevant activity, the children’s perception of current awareness was consciously guided to experience something different from their previous awareness, which could amplify their cognition and promote the development of EFs. Training was conducted four times a week for 40 min each time and a total of 12 times. After each course, specific homework tasks were assigned, including formal and informal training. Table 4 shows the training program used in the study of the experimental group.

### 3.2. Results

#### 3.2.1. Preintervention Homogeneity Test for the Experimental and Control Groups

The results of the independent sample t test are shown in Table 5. There was no significant difference between the scores of mindfulness and those of EF dimensions at the pretest level between the two groups, indicating that the experimental group and the control group were homogeneous in their preintervention levels of mindfulness and EFs.

#### 3.2.2. Effect of Mindfulness Training on the Mindfulness Level of Children in Grade 4

The Levene test of variance equivalence results showed *F*(1, 43) = 0.17, *p* > 0.05, meeting the conditions of the covariance test. The ANCOVA results are displayed in Table 6. The mindfulness scores of the post-test experimental group were significantly different from those of the control group (*F*(1, 42) = 7.46, *p* < 0.01, *η*^2^ = 0.15). The post-test mean mindfulness score showed that the mindfulness score of the experimental group was significantly higher than that of the control group, which indicated that mindfulness training significantly improved the subjects’ mindfulness level.

#### 3.2.3. Effect of Mindfulness Training on EFs in 4th-Grade Children

The descriptive statistics are presented in Table 7, and the ANCOVA results are shown in Table 8. In this study, the sample sizes of the two groups were essentially equivalent, and the impact of covariance differences on the results was minimal, allowing for the use of ANCOVA (Guo, 2015). All measures met the assumption of parallelism and were suitable for covariance analysis. The ANCOVA results revealed that a significant difference was observed in working memory scores between the experimental and control groups during the post-test (*F*(1, 42) = 5.28, *p* = 0.03, *η*^2^ = 0.12). Post-test comparisons revealed that the experimental group scored significantly higher than the control group *(t* = 2.30, *p* = 0.03, Cohen’s *d* = 0.72), indicating an improvement in working memory in the experimental group following the mindfulness intervention. There was no significant difference in the stop signal reaction times between the experimental and control groups during the post-test (*F*(1, 42) = 0.13, *p* = 0.72, *η*^2^ < 0.01), indicating no significant difference in inhibitory control between the two groups at the post-test level. Similarly, there was no significant difference in cognitive flexibility reaction times between the experimental and control groups during the post-test (*F*(1, 42) = 0.39, *p* = 0.54, *η*^2^ = 0.01), suggesting no significant difference in cognitive flexibility between the two groups at the post-test level. Additionally, there was no significant difference in interference inhibition scores between the experimental and control groups during the post-test (*F*(1, 42) = 0.15, *p* = 0.70, *η*^2^ < 0.01), indicating no significant difference in interference inhibition between the two groups at the post-test level. Overall, mindfulness training significantly enhanced working memory in 4th-grade rural Chinese students with general baseline mindfulness levels but did not improve cognitive flexibility or inhibitory control.

## 4. Discussion

This study investigated the relationship between mindfulness and EFs among rural Chinese elementary school students from both developmental and training perspectives while also developing and implementing an appropriate mindfulness training program to facilitate the growth of mindfulness and EFs. Study 1 confirmed the development of mindfulness and the relationship between mindfulness and EFs. Study 2 verified the effects of mindfulness training on mindfulness and working memory (a key component of EFs) in 4th-grade elementary school students through an experimental method, though the training had no significant effects on other EF components. A development of mindfulness in grades 4–5 of elementary school in rural China was observed. Children in grades 4–5 of rural elementary school who performed well in mindfulness also did well from the perspectives of emotional control, working memory, initiation, shifting, inhibition, planning, monitoring, and organization of materials. Finally, the short-term mindfulness training demonstrated a positive impact on working memory among 4th-grade rural Chinese children, while its effects on inhibitory control and cognitive flexibility were not statistically significant.

The findings of this study indicate that mindfulness develops among rural elementary school students during the 4th and 5th grades, a discovery that enriches our understanding of mindfulness. The development of mindfulness might be related to individual-directed attention, which helps humans focus on task-relevant stimuli in the presence of distracting stimuli (Wyatt & Machado, 2013). Several studies have shown that directional attention develops between six and ten years of age. When it is necessary to choose orientation, older elementary school students are more precise in scanning the perceptual field (Commodari, 2016), which might help with the formation and realization of mindfulness. Research has shown that the development of mindfulness significantly impacts individual development. Children with well-developed mindfulness are more likely to experience positive emotions and feel less stressed and anxious (Schonert-Reichl & Lawlor, 2010). As a result of focusing on the period during which children’s mindfulness develops rapidly and cultivating children’s mindfulness in a targeted manner, children can form positive attitudes toward the external environment early in life, which is very helpful for their physical and mental health.

Mindfulness requires the conscious maintenance of attention in the present state and nonjudgmental acceptance of thoughts that currently appear in the brain (Kabat-Zinn, 2003). This process is closely related to EFs (Lyvers et al., 2014). Both mindfulness and EFs of children of elementary school develop rapidly (Greenberg & Harris, 2012; Y. Wang et al., 2012), and a close connection between mindfulness and EFs was indeed observed. The results of this study showed that among children in grades 4–5 of elementary school, mindfulness was significantly related to EFs and its subdimensions. In other words, the more children’s mindfulness developed, the stronger their EFs was, consistent with previous findings (Geronimi et al., 2020). This study focused on grades 4–5 in elementary school. Mindfulness was significantly related to eight subdimensions of EFs—emotional control, working memory, initiation, shifting, inhibition, planning, monitoring, and organization of materials—which may be related to how mindfulness affects cognitive functions. Research by Zelazo and Lyons (2012) showed that mindfulness practices might simultaneously entail cognitive processes in areas related to the prefrontal cortex and subcortical areas of the brain. Studies have shown that mindfulness can activate cognitive functions related to emotional regulation and behavioral control (Chambers et al., 2009; Lyvers et al., 2014), which require monitoring (Pruessner et al., 2020), cognitive flexibility (Deng et al., 2023), and inhibition (Hsieh & Chen, 2017). In this study, the development of EFs did not show significant differences between the 4th and 5th grades in elementary school. This could be because children in the 4th and 5th grades are of the same age group, potentially resulting in minor differences in EFs (J. Wang et al., 2009). Additionally, the EFs of rural children may develop at a slower pace (Freitas et al., 2022). This also indicates that, compared to EFs, the development of mindfulness among 4th- and 5th-grade rural elementary school students is more sensitive. Therefore, it is advisable to initiate executive function training through mindfulness-based approaches.

In this study, mindfulness training was implemented for fourth-grade students in rural elementary schools who have room for development, aiming to explore the influence of mindfulness training in enhancing mindfulness and EFs. The results showed that mindfulness training increased the level of mindfulness in 4th-grade children and had positive effect on working memory, whereas its effects on cognitive flexibility and inhibitory control were not statistically significant. Previous studies have similarly shown that mindfulness training protects and enhances the working memory levels of high-demand cohorts and others (Jha et al., 2022; Mrazek et al., 2013). Mindfulness may be associated with working memory and plays a specific role in attentional processes. To some extent, the focus on present moment experiences involves the active maintenance and manipulation of information within working memory (Jha et al., 2019). Mindfulness training can help children enhance their present moment awareness, thereby improving working memory capacity.

Many studies indicate that mindfulness training exerts beneficial effects on EFs (Gallant, 2016; Lassander et al., 2020). However, the three subcomponents of EFs—cognitive flexibility, working memory, and inhibitory control—are interrelated yet independent (Miyake et al., 2000). Research may yield divergent findings depending on variations in study populations and methodological approaches. This study did not find a positive effect of mindfulness training on cognitive flexibility or inhibitory control. These findings align with some prior research. A study involving 9–10-year-old children reported that mindfulness practice improved “hot” inhibitory control (emotionally charged contexts) but not “cold” inhibitory control measured using paradigms comparable to our study (Rezende et al., 2023). Convergent evidence from a systematic review indicates that mindfulness interventions demonstrate the smallest effect sizes on cognitive flexibility compared to their impacts on working memory and inhibitory control (Cásedas et al., 2020). In our study, Study 1 assessed enduring mindfulness-related characteristics, likely reflecting consolidated psychological resources developed through prolonged life experiences that may broadly support EFs’ subcomponents. In contrast, Study 2’s mindfulness-based intervention transiently enhanced state mindfulness, with effect variations observed across three EF components potentially linked to their differential intervention sensitivity and temporal developmental patterns. Working memory undergoes rapid development around the age of 10 (Gathercole et al., 2004), the stage of rapid development of inhibitory control is relatively early, and cognitive flexibility increases to adult levels around the age of 15 (Huizinga et al., 2006). For 4th-grade elementary school students, inhibitory control and cognitive flexibility are not in a rapid developmental phase compared to working memory. Additionally, short-term mindfulness training modulates activation and functional connectivity in the DLPFC, a core neural substrate supporting working memory (Tang et al., 2015). Cognitive flexibility relies on the dynamic coordination of the ACC–parietal–prefrontal network, with the optimization of its functionality requiring sustained training protocols (e.g., task-switching exercises) to progressively establish neural adaptations through neuroplastic mechanisms (Dajani & Uddin, 2015). The enhancement of inhibitory control is closely associated with the plasticity of the ACC-basal ganglia circuitry, and the reorganization of such neural circuits necessitates sustained training over a period of several months or more to achieve optimal neuroadaptive modifications (Lutz et al., 2008). These may explain why the intensity of mindfulness training in this study has a limited impact on cognitive flexibility and inhibitory control. For rural children, the chronic stress they often face may also influence the development of EFs (Evans & Kim, 2013), with working memory being more sensitive to stress (Sliwinski et al., 2006). Mindfulness training may alleviate stress and thereby improve working memory in children. Rural children from low-SES backgrounds experience compromised prefrontal development due to resource constraints (Noble et al., 2015), while chronic stress impairs inhibitory control through HPA axis hyperactivity (Blair & Raver, 2015). According to MAT, mindfulness interventions may partially mitigate these effects by enhancing attentional monitoring (e.g., breath-focused training optimizing DLPFC functionality; Jha et al., 2010) and reducing emotional arousal (Segal et al., 2021). Mindfulness training may initially prioritize “filling in baseline deficits”, and inhibitory control and cognitive flexibility require higher-order self-regulation, making short-term breakthroughs of the “resource bottleneck” challenging. The specific mechanisms through which mindfulness buffers SES-related disadvantages require further empirical validation in future studies. Furthermore, a previous study noted that mindfulness training leads to fewer errors and longer reaction times in cognitive control tasks. This study concludes that mindfulness training can effectively enhance proactive control without significantly affecting reactive control, suggesting that mindfulness training may help individuals overcome interference (Y. Li et al., 2018). This aligns with the trends observed in our experimental results. Working memory tasks involve cognitive functions that individuals can actively control, whereas tasks measuring cognitive flexibility and inhibitory control primarily assess passive responses. This distinction may explain why mindfulness training did not significantly improve performance in the latter two domains.

The effects of mindfulness training on EFs are multifaceted, and the link between mindfulness and EFs is not straightforward. In Study 1, we observed an association between mindfulness and daily EFs performance. Study 2 further demonstrates that mindfulness training is more effective in enhancing working memory. The findings of this study on mindfulness training for 4th-grade rural elementary school students contribute to the growing body of research in this field and provide practical guidance for the development of training programs tailored to elementary school students. Because mindfulness training was conducted according to the established classroom time of Chinese elementary school students and the mindfulness training protocol was reproducible, the present study of such training can be extended more effectively to elementary schools of China or countries with similar course designs to help more students improve the quality of EFs.

This study has certain limitations. First, although we observed that components of EFs were more closely related to mindfulness and the direction of action of mindfulness training on EFs, the specific mechanism of the connection between mindfulness and each component of EFs was not covered in this study. Further exploration of this topic is needed; MAT posits that attentional monitoring and acceptance are fundamental mechanisms underlying the effects of mindfulness and mindfulness training and hypothesizes that the practice of attentional monitoring in mindfulness training interventions is a key mechanism for improving cognitive functioning outcomes in an emotionally neutral context (Lindsay & Creswell, 2017). Future empirical research could be conducted to validate the mechanisms by which mindfulness training occurs to work and incorporate initiation of emotion. For instance, this may involve incorporating additional variables and conducting further research or considering more nuanced categorizations of the types of mindfulness training or the characteristics of the participants. Second, future research could further refine intervention strategies. For instance, the curriculum could be combined with games to help children develop an awareness of mindfulness and bring such awareness into everyday life. The curriculum and exercises should not only comply with the rules of formal practice but also be relevant to children’s lives. In this sense, this study requires additional integration into real life. In addition, researchers have found that mindfulness ecological momentary interventions are effective, especially on longer-term outcomes (Zainal & Newman, 2024). Future research could also introduce brief mindfulness ecological momentary interventions to conduct concise interventions. Concurrently, as this study was conducted in rural China, mindfulness practices may require cultural adaptations given the variations documented across contexts. This underscores the imperative to refine interventions through culturally informed frameworks in future research while advancing cross-cultural comparative studies to elucidate mindfulness training’s contextualized mechanisms. Third, this study did not consistently track the effects of the intervention by examining the continued effectiveness of mindfulness training on EFs. Future research should supplement this aspect of measurement. Additionally, the current study found no significant effects of mindfulness training on inhibitory control or cognitive flexibility. Most mindfulness-based interventions included in-person weekly intervention sessions lasting 60–150 min for 8 weeks (e.g., MBCT and MBSR programs; Creswell, 2017). Future investigations should employ extended intervention durations to clarify mindfulness’s long-term impacts on these executive function components. Finally, methodological limitations warrant consideration. Study 1’s reliance on parent-reported EF measures may introduce potential reporting biases, underscoring the need for objective neurophysiological assessments (e.g., fMRI, EEG) in subsequent research. Due to the actual training process, only subjects with average mindfulness scores were selected. In the future, we should continue to explore the impact of mindfulness training on the mindfulness and EFs of children with strong mindfulness performance based on a larger sample.

## Figures and Tables

**Table 1:** Comparison of grade differences in mindfulness and EF in grades 4–5 (*n* = 346).

	Grade 4			Grade 5			*F*	*p*
	*n*	*M*	*SD*	*n*	*M*	*SD*		
Mindfulness	159	63.74	11.82	187	67.98	11.92	13.03	<0.01
EF	103	124.41	26.00	124	126.46	22.46	0.46	0.50

**Table 2:** Descriptive statistics of correlation analysis of mindfulness and EFs in grades 4–5 (*n* = 227).

	Mindfulness	*M*	*SD*
Mindfulness	1	67.15	12.07
Emotional Control	−0.27 **	14.11	3.37
Working Memory	−0.33 **	15.41	3.61
Initiate	−0.30 **	11.78	2.76
Organization of Materials	−0.26 **	8.89	2.51
Shift	−0.30 **	11.48	2.41
Plan/Organize	−0.28 **	18.06	4.16
Monitor	−0.28 **	13.01	3.10
Inhibit	−0.28 **	13.13	3.11
Executive Function	−0.34 **	125.56	24.11

** Significantly correlated at the 0.01 level.

**Table 3:** Stratified linear regression results for mindfulness and EF in grades 4–5 (*n* = 227).

	Executive Function		Emotional Control		Working Memory		Initiate		Organization of Materials		Shift		Plan/Organize		Monitor		Inhibit	
	1st Model *B(t)*	2nd Model *B(t)*	1st Model *B(t)*	2nd Model *B(t)*	1st Model *B(t)*	2nd Model *B(t)*	1st Model *B(t)*	2nd Model *B(t)*	1st Model *B(t)*	2nd Model *B(t)*	1st Model *B(t)*	2nd Model *B(t)*	1st Model *B(t)*	2nd Model *B(t)*	1st Model *B(t)*	2nd Model *B(t)*	1st Model *B(t)*	2nd Model *B(t)*
First Layer																		
Sex	−3.42 (−1.06)	−1.35 (−0.44)	0.70 (1.57)	0.95 * (2.25)	−0.38 (−0.78)	−0.09 (−0.20)	−0.41 (−1.12)	−0.21 (−0.59)	−0.35 (−1.04)	−0.19 (−0.58)	−0.08 (−0.24)	0.12 (0.39)	−0.67 (−1.21)	−0.39 (−0.73)	−0.69 (−1.68)	−0.48 (−1.20)	−0.58 (−1.40)	−0.37 (−0.92)
Grade	2.57 (0.68)	6.78 (1.88)	0.73 (1.41)	1.25 * (2.49)	0.08 (0.14)	0.67 (1.22)	0.27 (0.64)	0.69 (1.64)	0.19 (0.50)	0.52 (1.33)	0.69 (1.84)	1.08 ** (2.99)	0.24 (0.37)	0.81 (1.27)	0.46 (0.95)	0.89 (1.88)	0.26 (0.53)	0.69 (1.44)
Age	−0.51 (−0.24)	−1.56 (−0.79)	0.25 (0.85)	0.12 (0.42)	−0.05 (−0.16)	−0.20 (−0.66)	−0.07 (−0.31)	−0.18 (−0.77)	−0.17 (−0.76)	−0.25 (−1.16)	−0.16 (−0.76)	−0.26 (−1.29)	−0.23 (−0.65)	−0.38 (−1.07)	−0.30 (−1.11)	−0.40 (−1.56)	−0.15 (−0.57)	−0.26 (−1.00)
Second Layer																		
Mindfulness		−0.73 ** (−5.70)		−0.09 ** (−5.06)		−0.10 ** (−5.26)		−0.07 ** (−4.81)		−0.06 ** (−4.05)		−0.07 ** (−5.30)		−0.10 ** (−4.37)		−0.08 ** (−4.46)		−0.07 ** (−4.41)
Adjusted *R*^2^	−0.01	0.12	0.02	0.12	−0.01	0.10	−0.01	0.09	−0.01	0.06	0.00	0.11	−0.00	0.07	0.01	0.08	−0.00	0.07
*F*	0.54	8.59 **	2.68	8.62 **	0.22	7.09 **	0.56	6.25 **	0.58	4.56 **	1.16	8.01 **	0.65	5.30 **	1.46	6.16 **	0.81	5.51 **

** Significantly correlated at the 0.01 level. * Significantly correlated at the 0.05 level.

**Table 4:** Mindfulness training program for elementary school students in grade 4.

Stage	Class Schedule	Course Introduction
Awareness of Breathing and Sensory Practice Stage	First Week	Breathing: focus, balance, pause
		Listen to the sound and do the action
		Memory card
		Mini-game: open your ears
		Breathing rhythm
		Share and reflect
		Breathing: focus, balance, pause
		Memory card
		What did you hear?
		Mini-game: fierce eyes
		Breathing rhythm
		Share and reflect
		Breathing: focus, balance, pause
		Sensory switching
		Mini-game: brain imaging
		Breathing rhythm
		Share and reflect
		Homework: be mindful of your surroundings Homework: quiet breathing for 5 min Small challenge: commit to performing a mindfulness exercise every day
Awareness of Body Movement	Second Week	Breathing: focus, balance, pause
		Body scan
		Role playing: Jungle adventure
		Mini-game: be aware of feelings
		Breathing rhythm
		Share and reflect
		Breathing: focus, balance, pause
		Pendulum movement
		Mini-game: hand left, foot right
		Breathing rhythm
		Share and reflect
		Breathing: focus, balance, pause
		Balancing act
		Body scan
		Mindful eating
		Breathing rhythm
		Share and reflect
		Homework: eat mindfully Small challenge: commit to performing a mindfulness practice every day Small challenge: use mindfulness to experience everyday things
Awareness of the Mind	Third Week	Breathing: focus, balance, pause
		Awareness of thoughts
		Shape space
		Mini-game: naming feelings
		Breathing rhythm
		Share and reflect
		Breathing: focus, balance, pause
		Sharing kindness
		Convey kindness
		Mini-game: spiritual journey
		Breathing rhythm
		Share and reflect
		Homework: breathing or body scan exercises Small challenge: use mindfulness to do something you find difficult

**Table 5:** Pretest of independent samples *t* test for each dimension of mindfulness and EF in the experimental and control groups (*n* = 45).

	Experimental Group		Control Group		*t*	*p*	Cohen’s *d*
	*M*	*SD*	*M*	*SD*			
Accuracy of Response Inhibition	0.56	0.13	0.53	0.14	0.60	0.55	0.18
Reaction Time of Response Inhibition	327.80	74.26	331.17	97.32	−0.13	0.90	0.04
Interference Inhibition	2.15	50.77	−9.42	51.98	0.75	0.46	0.22
Working Memory	9.36	3.48	9.00	3.43	0.35	0.73	0.10
Accuracy of Cognitive Flexibility	0.49	0.10	0.49	0.07	−0.02	0.99	0.01
Reaction Time of Cognitive Flexibility	492.67	223.53	370.78	223.01	1.83	0.07	0.55
Mindfulness	58.10	9.59	56.63	7.06	0.59	0.56	0.18

**Table 6:** ANCOVA of mindfulness post-test scores for the experimental and control groups (*n* = 45).

	Experimental Group		Control Group		*F*	*p*	*η* ^2^
	*M*	*SD*	*M*	*SD*			
Mindfulness	68.10	9.28	59.79	10.34	5.55	0.02	0.12

**Table 7:** Descriptive statistics for pre- and post-test measures of the experimental and control groups (*n* = 45).

		Experimental Group		Control Group	
		*M*	*SD*	*M*	*SD*
Mindfulness	Pre	58.10	9.59	56.63	7.06
	Post	68.10	9.28	59.79	10.34
Accuracy of Response Inhibition	Pre	0.56	0.13	0.53	0.14
	Post	0.66	0.19	0.58	0.17
Reaction Time of Response Inhibition	Pre	327.80	74.26	331.17	97.32
	Post	368.90	93.48	356.08	118.43
Reaction Time of Interference Inhibition	Pre	2.15	50.77	−9.42	51.98
	Post	26.87	56.57	16.63	36.45

**Table 8:** ANCOVA of post-test scores for the experimental and control groups (*n* = 45).

	Response Inhibition			Cognitive Flexibility			Interference Inhibition			Working Memory		
	*F*	*p*	*η* ^2^	*F*	*p*	*η* ^2^	*F*	*p*	*η* ^2^	*F*	*p*	*η* ^2^
Group	0.13	0.72	0.00	0.39	0.54	0.01	0.15	0.70	0.00	5.28	0.03 *	0.12
Pretest	16.18	0.00 **	0.29	12.70	0.00 **	0.25	0.45	0.50	0.01	3.11	0.09	0.07
Accuracy	11.30	0.00 **	0.22	35.58	0.00 **	0.48						
Sex	1.13	0.29	0.03	2.12	0.15	0.05	0.00	0.98	0.00	1.25	0.27	0.03
Age	0.09	0.77	0.00	2.29	0.14	0.06	0.45	0.51	0.01	0.18	0.67	0.00

** Significantly correlated at the 0.01 level. * Significantly correlated at the 0.05 level.

## Data Availability

The raw data supporting the conclusions of this article will be made available by the authors on request.

## Abbreviations

The following abbreviations are used in this manuscript:

EF	Executive function
ANCOVA	Analysis of covariance
MAT	Monitor and Acceptance Theory
ACC	Anterior cingulate cortex
DLPFC	Dorsolateral prefrontal cortex
SES	socioeconomicstatus
